# The Impact of Expectation Management and Model Transparency on Radiologists’ Trust and Utilization of AI Recommendations for Lung Nodule Assessment on Computed Tomography: Simulated Use Study

**DOI:** 10.2196/52211

**Published:** 2024-03-13

**Authors:** Lotte J S Ewals, Lynn J J Heesterbeek, Bin Yu, Kasper van der Wulp, Dimitrios Mavroeidis, Mathias Funk, Chris C P Snijders, Igor Jacobs, Joost Nederend, Jon R Pluyter, e/MTIC Oncology group

**Affiliations:** 1 Catharina Cancer Institute, Catharina Hospital Eindhoven Eindhoven Netherlands; 2 Department of Experience Design, Royal Philips Eindhoven Netherlands; 3 Research Center for Marketing and Supply Chain Management, Nyenrode Business University Breukelen Netherlands; 4 Department of Data Science, Philips Research Eindhoven Netherlands; 5 Department of Industrial Design, Eindhoven University of Technology Eindhoven Netherlands; 6 Department of Human Technology Interaction, Eindhoven University of Technology Eindhoven Netherlands; 7 Department of Hospital Services and Informatics, Philips Research Eindhoven Netherlands; 8 See acknowledgments Eindhoven Netherlands

**Keywords:** application, artificial intelligence, AI, computer-aided detection or diagnosis, CAD, design, human centered, human computer interaction, HCI, interaction, mental model, radiologists, trust

## Abstract

**Background:**

Many promising artificial intelligence (AI) and computer-aided detection and diagnosis systems have been developed, but few have been successfully integrated into clinical practice. This is partially owing to a lack of user-centered design of AI-based computer-aided detection or diagnosis (AI-CAD) systems.

**Objective:**

We aimed to assess the impact of different onboarding tutorials and levels of AI model explainability on radiologists’ trust in AI and the use of AI recommendations in lung nodule assessment on computed tomography (CT) scans.

**Methods:**

In total, 20 radiologists from 7 Dutch medical centers performed lung nodule assessment on CT scans under different conditions in a simulated use study as part of a 2×2 repeated-measures quasi-experimental design. Two types of AI onboarding tutorials (reflective vs informative) and 2 levels of AI output (black box vs explainable) were designed. The radiologists first received an onboarding tutorial that was either informative or reflective. Subsequently, each radiologist assessed 7 CT scans, first without AI recommendations. AI recommendations were shown to the radiologist, and they could adjust their initial assessment. Half of the participants received the recommendations via black box AI output and half received explainable AI output. Mental model and psychological trust were measured before onboarding, after onboarding, and after assessing the 7 CT scans. We recorded whether radiologists changed their assessment on found nodules, malignancy prediction, and follow-up advice for each CT assessment. In addition, we analyzed whether radiologists’ trust in their assessments had changed based on the AI recommendations.

**Results:**

Both variations of onboarding tutorials resulted in a significantly improved mental model of the AI-CAD system (informative *P*=.01 and reflective *P*=.01). After using AI-CAD, psychological trust significantly decreased for the group with explainable AI output (*P*=.02). On the basis of the AI recommendations, radiologists changed the number of reported nodules in 27 of 140 assessments, malignancy prediction in 32 of 140 assessments, and follow-up advice in 12 of 140 assessments. The changes were mostly an increased number of reported nodules, a higher estimated probability of malignancy, and earlier follow-up. The radiologists’ confidence in their found nodules changed in 82 of 140 assessments, in their estimated probability of malignancy in 50 of 140 assessments, and in their follow-up advice in 28 of 140 assessments. These changes were predominantly increases in confidence. The number of changed assessments and radiologists’ confidence did not significantly differ between the groups that received different onboarding tutorials and AI outputs.

**Conclusions:**

Onboarding tutorials help radiologists gain a better understanding of AI-CAD and facilitate the formation of a correct mental model. If AI explanations do not consistently substantiate the probability of malignancy across patient cases, radiologists’ trust in the AI-CAD system can be impaired. Radiologists’ confidence in their assessments was improved by using the AI recommendations.

## Introduction

### Background

Lung cancer is one of the leading causes of cancer-related deaths worldwide [1]. Early detection of lung cancer is essential to provide curative treatment and improve survival. However, detecting and diagnosing lung cancer using computed tomography (CT) scans can be challenging. On CT scans, early lung cancer can be seen as a small nodule. However, these nodules can also be benign. The risk of malignancy depends on various patient factors and lung nodule features, such as the morphology, size, and number of lung nodules. Nodules that are challenging to detect can, for instance, be small, and their perceptibility might be hampered by their location close to normal lung tissue that is visually similar on a CT scan, such as blood vessels or bronchi [2-5]. As a result, radiologists may overlook or misdiagnose lung nodules on CT scans. A previous study showed that radiologists missed 15% of all lung cancer cases on screening CT scans. Of these missed cancers diagnoses, 35% were not visible on the scan, 50% were not detected by the radiologist, and 15% were detected but not diagnosed as cancer [6].

A recent approach to improve the detection and diagnosis of lung nodules on CT scans is the use of artificial intelligence (AI) models. Diagnostic assistance from AI models that provide recommendations for radiologists is referred to as AI-based computer-aided detection or diagnosis (AI-CAD) [7]. Many studies have been published on AI models for assessing lung nodules on CT scans, showing promising performance with sensitivities for detection of up to 98.1% and a mean of only 2 false-positives (FPs) per scan [8,9].

Although many AI models and AI-CAD systems have been developed, few are used in clinical practice. Although most studies on AI for lung nodule assessment focus on the development and stand-alone performance of AI models [8,10,11], few studies have focused on user interaction with AI models in the clinical context beyond the theoretical level [12-16]. However, human-AI interaction is essential to enable radiologists to comprehend and effectively use AI recommendations in their tasks, ultimately achieving the highest levels of diagnostic quality and efficiency.

Trust is of great importance in the interactions and collaborations between radiologists and AI-CAD systems [15,17-20]. Trust influences the end users’ level of reliance on AI recommendations, and hence, it influences the performance of AI-assisted end users [18,19]. If the user has very little trust in the system, the potential benefits of AI-CAD will be reduced because of disuse, whereas too much trust in the system leads to overreliance and can result in mistakes that would not have been made without using the AI-CAD system [15,18].

Trust is a dynamic process. Trust changes over time and across situations and is influenced by many factors. For example, trust varies based on the reliability of the AI system, the design of the system, the personal characteristics of the user, prior interactions and experience, and moderating factors such as workload and sociocultural context [18,21-25]. Some of these factors can be influenced through the design of the system, with the aim of achieving the formation of appropriate trust. Trust calibration refers to interventions that facilitate the formation of an appropriate trust level by aligning a person’s trust in the AI with the capabilities of the AI [26,27]. In this study, we introduced 2 instruments aimed at appropriate trust calibration at different time points of use. First, an onboarding tutorial aimed to set the right expectations before initial use. Second, AI model explainability as an information cue available to clinical users during use to judge the credibility of the arguments underpinning the AI model prediction.

We aimed to assess whether radiologists’ trust in AI-CAD systems and their use of AI recommendations in lung nodule assessments on CT scans were affected by different onboarding tutorials and by different levels of AI model explainability.

### Theoretical Argumentation

#### Trust Definitions

Different definitions and measures exist for trust [15]. In this study, we considered trust from 2 complementary perspectives, a cognitive perspective and a behavioral perspective [23].

From the cognitive perspective, we explored the users’ mental model and psychological trust. The *mental model* represents a person’s “static knowledge about the system: its significant features, how it functions, how different components affect others, and how its components will behave when confronted with various factors and influences” [24]. In short, the mental model is the user’s understanding of the AI system. A correct mental model is expected to contribute to appropriate trust calibration between the user’s trust in an AI system and the trustworthiness of the system [25]. User’s *psychological trust* refers to “the extent to which a user is confident in, and willing to act on the basis of, the recommendations, actions, and decisions of an artificially intelligent decision aid” [28]. Because radiologists gain experience and learn through the process of assessing CT cases with the AI-CAD tool and actually see what the system is capable of, they are expected to have an improved mental model of (hypothesis 1a) and psychological trust in (hypothesis 1b) the AI-CAD system after using the AI-CAD system compared with before using the system.

However, holding a positive attitude toward the AI-CAD system does not mean that the user will also act in line with its recommendations. Therefore, we also adopted a behavioral lens by examining whether trust was reflected in the *use of the AI recommendations* (reliance and compliance) and the corresponding impact on decision outcomes [29,30]. The decision of whether radiologists use AI recommendations depends not only on their overall trust in the AI-CAD system but also on their agreement with the specific AI recommendations for a given case. As the AI recommendations function as a second reader, it is expected that radiologists’ confidence in their assessments will be higher when they are assisted by AI-CAD than without assistance (hypothesis 2).

#### Onboarding Tutorials

Research on how to ensure that radiologists have appropriate expectations of the system’s capabilities and limitations is limited [27]. As suggested by Cai et al [31], when clinical practitioners are first introduced to an AI system, a human-AI onboarding process can be crucial for them to determine how they will partner with AI in practice. Therefore, an onboarding tutorial to inform radiologists about the capabilities and limitations of the AI-CAD system is expected to improve radiologists’ mental model of (hypothesis 3a) and psychological trust in the AI-CAD system (hypothesis 3b).

Moreover, critical reflection on one’s experience is essential for developing competence and self-awareness [32]. Hence, it is hypothesized that critical reflection and feedback built through a reflective onboarding tutorial will lead to a more improved mental model of (hypothesis 4a) and psychological trust in (hypothesis 4b) the AI-CAD system than an informative onboarding tutorial. Furthermore, it is expected to be easier for radiologists to understand whether an AI suggestion should be followed because of their understanding of the AI-CAD system from reflective onboarding, especially when they are not fully sure of their own assessment. Therefore, it is expected that radiologists who receive reflective onboarding will use the AI recommendations more often than radiologists who receive informative onboarding (hypothesis 5).

#### Levels of AI Model Explainability

In addition, radiologists are expected to better judge whether they can trust an AI recommendation when the AI model discloses the reasoning behind its recommendations (explainable AI models) compared with black box models. Hence, it is hypothesized that after using the AI-CAD system, radiologists assisted with explainable AI output have an improved mental model of (hypothesis 6a) and psychological trust in (hypothesis 6b) the AI-CAD system than radiologists assisted with black box AI output. Because radiologists can see the reasoning behind the recommendations when receiving explainable AI output, it is expected that they will use the AI recommendations more often than radiologists assisted with black box AI output (hypothesis 7).

## Methods

### Overview

We tested the hypotheses using a 2×2 repeated-measures quasi-experimental design: informative versus reflective onboarding tutorial and black box versus explainable AI output. In this simulated use study, we aimed to realistically mimic clinical practice [33,34]. Realistic clinical simulations allow participants to engage with the setup in real-world clinical scenarios and encourage participants to authentically execute the study as if they are performing their clinical work.

### Prototype

#### Image Viewer

**Figure 1:**
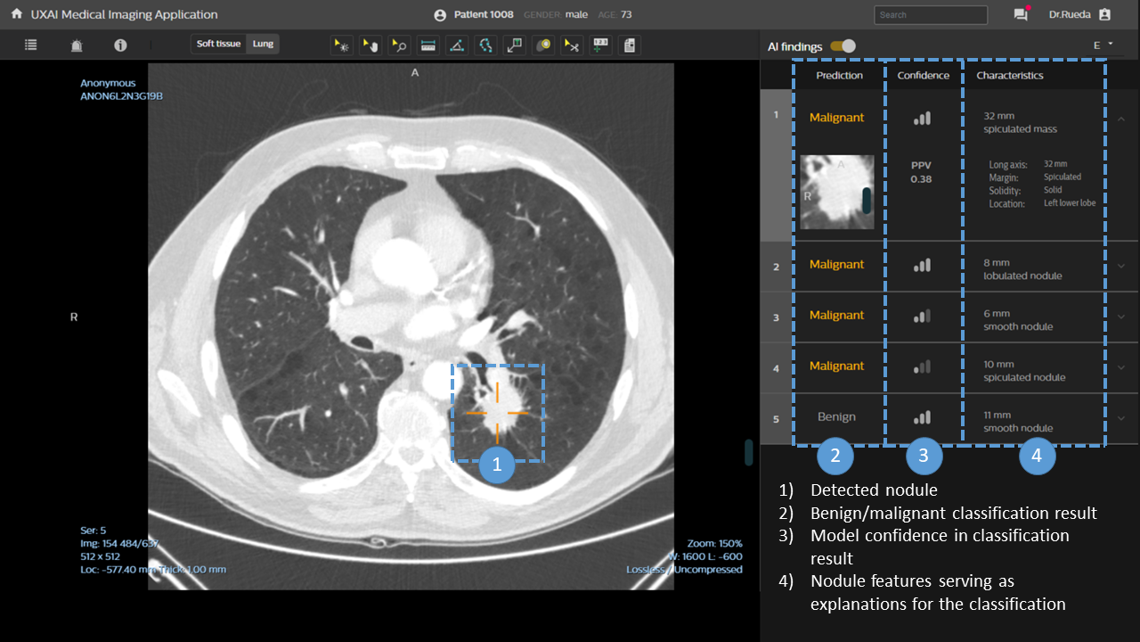
Medical image–viewing prototype in the explainable artificial intelligence (AI) condition. In the black box AI condition, users could not see the nodule characteristics column on the right side of the screen. When the AI findings toggle is off, all AI recommendations will be hidden to the users.

A medical image–viewing prototype was developed to enable radiologists to assess incidental lung nodules on cardiac CT scans with and without the assistance of an AI-CAD system. The AI recommendations were implemented as a second reader, allowing the radiologist to first assess the cases independently. The interface was designed based on the literature, brainstorms, and feedback sessions with radiologists and design specialists and was iteratively optimized for the 2 variations of onboarding tutorials (reflective vs informative) and 2 variations of AI outputs (black box vs explainable). The final user interface is shown in Figure 1. We aimed to realistically simulate the radiologists’ clinical setup to facilitate proper engagement of the participants with the task of lung nodule assessment. The user setup was designed to simulate clinical practice as realistically as possible. The developed interface was shown to the radiologists on a monitor, which was placed in a separate silent room. This room was inside the hospital, and lights could be dimmed if the radiologists preferred it, comparable with their own working space. Similar to the picture archiving and communication system used in clinical practice to assess CT scans, radiologists could scroll through the images, zoom in, measure, and change the windowing level between the soft tissue and lung setting using a computer mouse.

#### Clinical Data

To further increase study engagement and realism, the use scenarios were based on real-world patient cases. We retrospectively selected 10 CT angiography scans with incidental pulmonary nodules from a large Dutch clinical hospital. Scans acquired between 2008 and 2015 were used because the 5-year outcomes of these patients are known: whether they developed lung cancer. An expert radiologist selected the cases for this study. Of the 10 selected scans, we used 3 for onboarding and 7 for testing the impact of the design interventions. All CT scans were performed on patients with lung cancer. By selecting the 7 CT cases, we aimed to obtain a diverse mix of assessment complexity by including both lower and higher suspicious nodules (based on size, spiculation, and solidity) and nodules at easier and more difficult locations (such as against the veins or pleura). The characteristics of the 7 CT cases and the findings of the AI model for these cases are presented in Multimedia Appendix 1.

#### AI Model

To detect and estimate the malignancy of lung nodules on the CT scans, the pretrained AI framework developed by Trajanovski et al [35] was applied. This framework relies on a 2-stage process, where the first stage performs nodule detection and the second stage assigns a malignancy probability to the detected nodules. Among the validated nodule detectors, the best performance was achieved by the nodule detector developed by Liao et al [36]. This nodule detector is based on deep learning models, more precisely, convolutional neural networks. The nodules detected by the nodule detector are provided as input to the second stage of the framework that assigns the cancer malignancy probabilities. The second stage of the framework is based on a convolutional neural network that was trained using the publicly available National Lung Screening Trial data set [37].

During inference, the model takes a CT scan as input and automatically produces a list of nodule locations (x,y,z), their radii, and malignancy probabilities. The prototype, described previously, ensures that this information is displayed intuitively to the clinicians. The article by Liao et al [36] provides all the relevant details regarding the training process and performance validation.

In this study, the AI model proposed by Trajanovski et al [35] was used without any additional fine-tuning. Specifically, the model weights remained unchanged. The sole adjustment involved calibrating (or rescaling) the output of the model to accommodate the changed distribution of malignant cases (Multimedia Appendix 2 [35,38,39]).

#### AI Recommendations

The AI model recommendations were provided using 4 information cues (Multimedia Appendix 3):

1. Detected nodules (shown by target mark directly on the CT scan)
2. Benign or malignant classification per nodule (malignant nodules are highlighted in orange color)
3. Model confidence in the benign or malignant classification (shown as the negative predictive value [NPV] or positive predictive value [PPV] score and an intuitive icon representing high, medium, or low confidence)
4. In the explainable AI output variant: nodule features serving as an explanation for the classification

AI nodule detection and benign or malignant classification (cues 1 and 2) were obtained using the described AI model [35]. The number of lung nodules detected by the AI model varied between 1 and 5 per scan. The AI model found at least one true-positive lung nodule in each case and found one or more FP nodules in 4 of 7 cases. For more information about the AI findings, see Table S1 in Multimedia Appendix 1.

Confidence in the malignancy classification (cue 3) was given by means of PPVs for malignant predictions, indicating the probability that nodules with malignant predictions were actually malignant, and by means of NPVs for benign predictions, indicating the probability that nodules with a benign prediction were actually benign. The PPV was 0.25 (low confidence), 0.30 (medium confidence), or 0.38 (high confidence), and the NPV was 0.94 (low confidence), 0.97 (medium confidence), or >0.99 (high confidence; for an explanation of how the PPVs and NPVs were calculated, see Multimedia Appendix 2). In addition, confidence was shown by means of a small bar graph, indicating low, medium, or high model confidence.

Two levels of AI transparency were tested: black box AI output and explainable AI output. Black box output indicates that radiologists did not see what the malignancy estimation was based on. The explainable AI output variant provided the same information as the black box AI output variant and additionally showed the characteristics of the lung nodules (cue 4); this information was expected to help in understanding and interpreting the predictions of the AI-CAD system (Figure 1, right column). For each lung nodule, the following lung nodule characteristics were provided: long axis diameter, solidity, margin characteristics, and location. The nodule characteristics were not provided by the AI model and were therefore realistically simulated, which is in agreement with related research [40] via manual annotation by 2 expert radiologists in consensus. However, the participants were not aware of the simulation; therefore, from the radiologists’ perspective, the characteristics were AI generated as well [41]. For an overview of the information cues for the AI recommendations, see Multimedia Appendix 3.

#### Onboarding Tutorials

**Figure 2:**
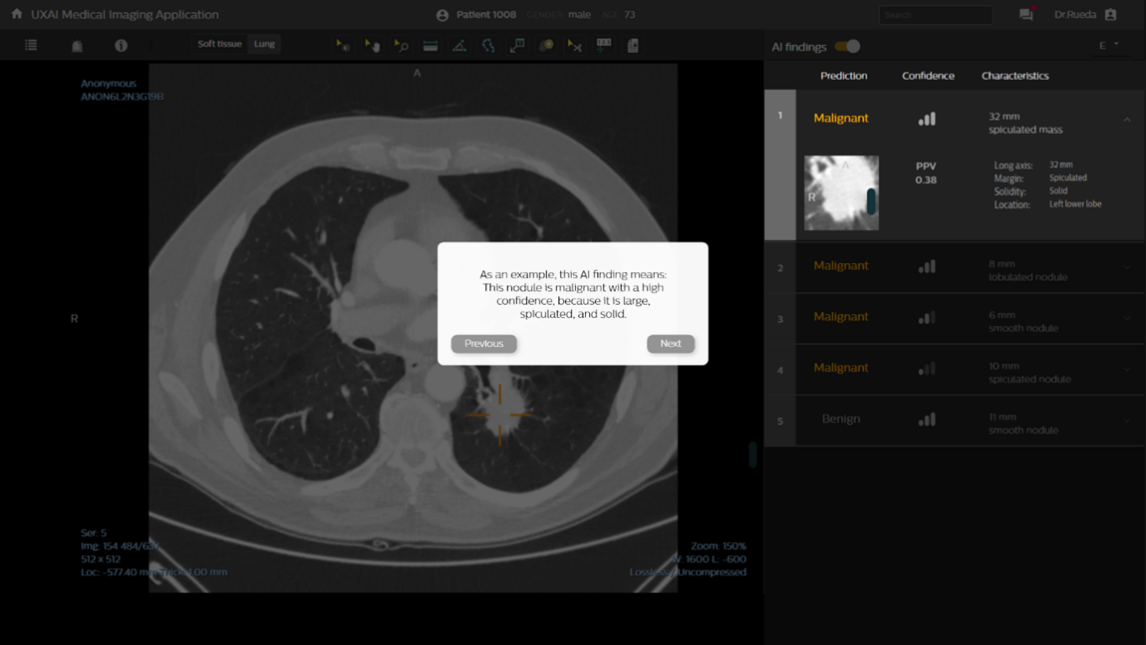
Onboarding tutorial in the informative onboarding condition, which provided a stepwise introduction of artificial intelligence (AI) capabilities and limitations using example patient cases. In the reflective onboarding condition, an additional question-answer dialog was triggered to provide feedback on whether the user’s expectations of the AI capabilities and limitations were correct.

Two variations of onboarding tutorials were designed: informative onboarding and reflective onboarding. During informative onboarding, radiologists passively received a stepwise introduction of the AI capabilities and common pitfalls so that they could acquire a realistic mental model of the system (Figure 2). The AI model’s capabilities and pitfalls were illustrated in the onboarding tutorial with 3 CT scans that showed obvious cancer cases, FP nodules, and false-negative nodules. For an overview of all implemented questions and explanations, see Multimedia Appendix 3. During reflective onboarding, radiologists additionally engaged in active reflection. They received cognitive feedback on 4 questions that they had to answer to check whether their mental model of the AI-CAD system was correct.

### Study Protocol

**Figure 3:**
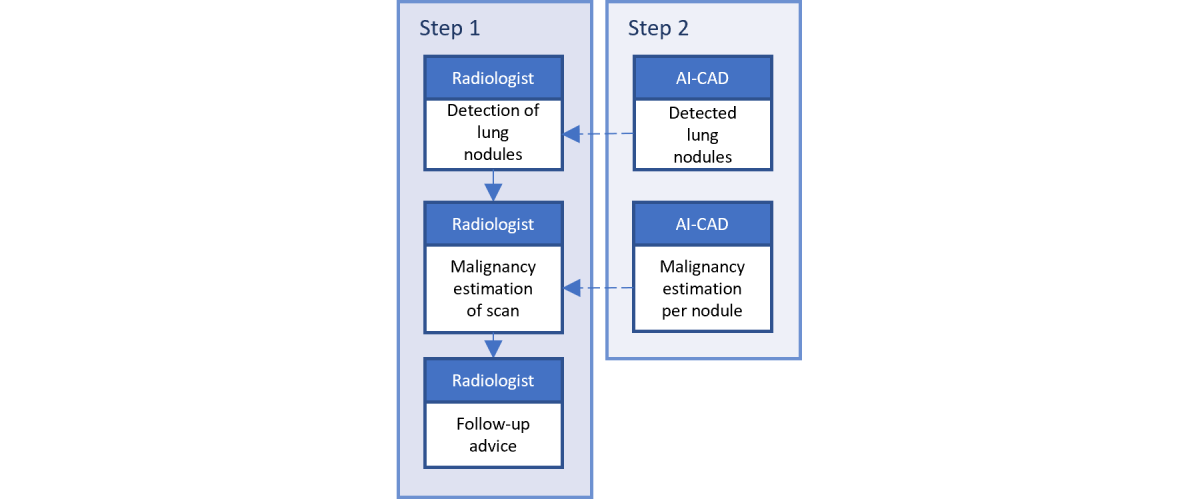
Flow diagram showing the clinical decisions of radiologists, which might potentially be influenced by the outcomes of the artificial intelligence model. The detected nodules may influence the malignancy estimation, and the malignancy estimation may influence the follow-up advice. AI-CAD: artificial intelligence–based computer-aided detection or diagnosis.

For this study, physicians were eligible for participation if they were radiologists, nuclear radiologists, or radiology residents. We will refer to the participants as *radiologists*. Several effects were to be tested; we used a power of 80%. For the mental model differences between radiologists, we based our sample size calculation on a comparison of means of 2 versus 3 (SD 0.5). This led to a necessary sample size of 12 radiologists. For the psychological trust differences, we based the sample size calculation on a comparison of means of 0.5 versus 0.75 (SD 0.1). This resulted in a sample size of at least 8 radiologists. The differences in the use of AI recommendations were based on a comparison of proportions in the order of magnitude of 30% versus 10%. This leads to a necessary sample size of 124 comparisons if we assume that the intraclass coefficient is low. Eventually, 20 radiologists were included in this study, all of whom assessed 7 CT scans for a total of 140 recommendations [42]. In this 2×2 repeated-measures design, the radiologists were divided into 4 groups, each of which consisted of 5 radiologists. After onboarding in one of the 2 conditions, using 3 CT scans, each radiologist assessed the 7 CT scans. In addition to the CT scans, each patient’s age and gender were provided because radiologists also use the patient context when they assess CT scans in clinical practice. First, the radiologists assessed the scans without observing the AI output. They reported the nodules they detected, estimated the malignancy probability for the patient case (not per nodule, unlike the AI model), and provided follow-up advice. Subsequently, the AI recommendations were presented, and the radiologists could adjust their initial assessments. The nodules detected by AI and the AI malignancy estimations might trigger the radiologists to change their initial assessments. This process is visualized in the flow diagram in Figure 3.

### Measures for Trust

**Figure 4:**
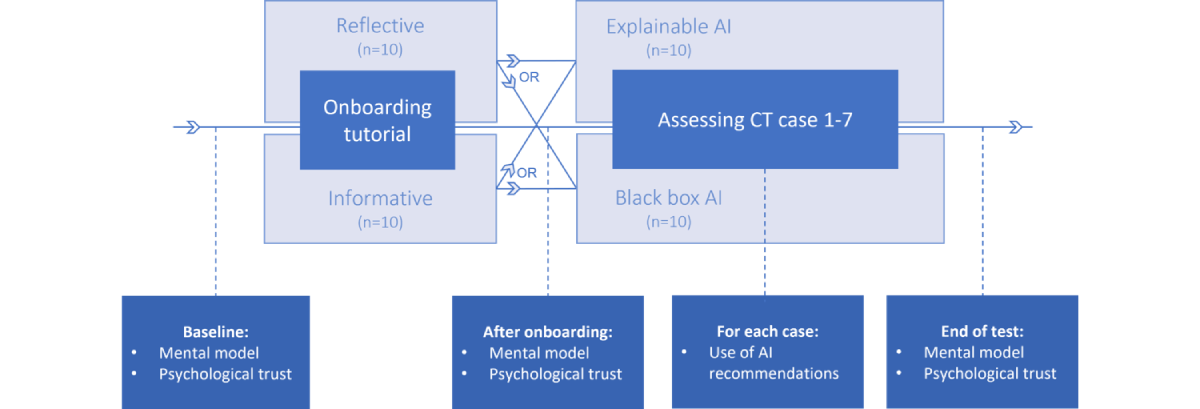
Overview of the flow of the experiment with the questionnaires at different time points. AI: artificial intelligence; CT: computed tomography.

To evaluate the effects of the 2 types of AI onboarding tutorials and the 2 levels of explainability of AI outputs on radiologists’ trust in AI and their use of AI recommendations, participants were requested to complete questionnaires on 3 aspects: the radiologists’ mental model of the AI-CAD system’s capabilities and pitfalls, psychological trust in the AI-CAD system, and the use of AI recommendations. These questionnaires were completed at different time points, as schematically shown in Figure 4.

#### Mental Model

The mental model questionnaire measured the radiologists’ understanding of the AI capabilities and limitations to uncover whether their expectations of the AI-CAD system were appropriate. Of the 11 questions in this questionnaire, 5 questions were related to nodule detection and 6 were related to malignancy prediction (see the full questionnaire in Multimedia Appendix 4). Questions could be answered with *yes*, *no*, or *I do not know*. Depending on whether the assessment was correct as compared with the true AI capabilities, a score of 1 (correct) or 0 (incorrect or *I do not know*) was assigned per question, resulting in summed scores between 0 and 11. A higher score implies a better understanding of the AI capabilities. The mental model was measured before onboarding, after onboarding, and after assessing the 7 CT scans.

#### Psychological Trust

To measure the radiologists’ psychological trust in the AI-CAD system, a questionnaire was derived from the study by Ashoori and Weisz [43] and adapted to fit this study (see the full questionnaire in Multimedia Appendix 4). This questionnaire examined overall trustworthiness, reliability, technical competence, and personal attachment. An example of a statement is “This model is trustworthy.” The 12 statements about the AI model had to be answered with a score between 1 (strongly disagree) and 5 (strongly agree). For the negatively phrased questions, scores were reversed for the data analysis so that for all questions, a higher score reflected more trust in the AI-CAD system. Subsequently, the scores for the 12 questions were averaged. The psychological trust of each participant was measured before onboarding, after onboarding, and after assessing the 7 CT scans.

#### Use of AI Recommendations

To evaluate the radiologists’ use of the AI recommendations, their assessments and confidence in their assessments—first without and then with AI assistance—were recorded in a questionnaire. AI recommendation use was measured at 3 assessment levels: number of detected nodules, malignancy probability, and follow-up advice. Therefore, the questionnaire included questions about the number of found nodules, the malignancy probability (at the patient level) as a percentage, and the follow-up advice according to the Fleischner guidelines [44]. The follow-up advice had to be scored with a score of 1 (consider CT at 3 months, positron emission tomography–CT, or tissue sampling), 2 (CT at 3-6 months), 3 (CT at 6-12 months), 4 (CT at 12 months), or 5 (no routine follow-up). A lower score indicated earlier follow-up. In addition, the confidence of the given answers at each assessment level had to be rated with a score between 1 (not confident at all) and 5 (very confident). The complete questionnaire is provided in Multimedia Appendix 4. Participants were requested to complete this questionnaire while assessing without AI assistance and with AI assistance for each CT case.

### Analyses

#### Mental Model and Psychological Trust

Changes in the mental model and psychological trust were assessed by comparing the scores before and after onboarding, and the scores after onboarding and at the end of the test, that is, after assessing all 7 CT scans. These changes were assessed for all radiologists together, for the 2 onboarding tutorial groups separately, and for the 2 AI output groups separately. The changes in scores were compared between the 2 onboarding tutorial groups and between the 2 AI output groups to analyze whether the types of onboarding tutorials and level of AI explainability influenced radiologists’ initial trust and maintenance of trust during CT assessment. In addition, we analyzed whether the changes in mental model and psychological trust scores were influenced by any of the following characteristics of the radiologists: age, gender, years of experience, how often they assessed lungs on CT as part of their job, how eager they were to try new information technologies, and how frequently they used AI-CAD tools.

#### Use of AI Recommendations

The use of AI recommendations was assessed by analyzing the number of cases in which radiologists adjusted the number of found nodules, the malignancy probability, and the follow-up advice after viewing the AI-CAD recommendations. In addition, we analyzed whether the radiologist’s confidence in the assessments of the number of nodules, the malignancy prediction, and the follow-up advice changed after viewing the AI recommendations and whether their confidence increased or decreased. The use of AI recommendations and the impact on radiologists’ confidence were compared between the groups of onboarding tutorials and between the groups of AI output.

### Secondary Analyses

#### Additional Analyses and Use of AI Recommendations

In addition, the impact of agreeing or disagreeing with the AI detected nodules was evaluated. We analyzed whether the use of AI recommendations and radiologists’ confidence in their assessments were affected by 2 factors: first, whether the same or different nodules were found by the AI as compared with the radiologist and, second, whether the radiologist changed the number of reported nodules after seeing the AI recommendations.

#### Correctness of Follow-Up Advice

Furthermore, to evaluate whether AI-CAD assistance resulted in improved clinical assessment, we analyzed whether the radiologists selected the correct follow-up advice more often with or without the AI recommendations. For each case, the correct follow-up according to the Fleischner criteria was retrospectively determined by 2 expert radiologists in consensus and used as reference follow-up advice. The follow-up recommendations provided by the radiologists were compared with the reference follow-up advice, and we analyzed whether AI assistance resulted in more accurate follow-up advice.

### Statistical Analyses

#### Mental Model and Psychological Trust

Differences between the mental model scores and psychological trust scores of the radiologists at different time points were analyzed using the Wilcoxon signed rank test. Differences between the mental model scores and psychological trust scores of the groups with informative and reflective onboarding tutorials and of the groups with black box and explainable AI output were statistically analyzed using Mann-Whitney *U* tests. To control for heterogeneity, we tested whether radiologists’ characteristics influenced the mental model scores and psychological trust scores at different time points and over time by performing multiple linear regression analyses.

#### Use of AI Recommendations

Multilevel logistic regression analyses were performed to assess whether the type of onboarding tutorial or level of explainability of the AI output influenced the use of the AI recommendations and the radiologists’ confidence in their assessments. To control for potential impact on the outcomes by other factors (exclusively the same nodules found by radiologists and AI model, change in number of reported nodules, age, gender, years of experience, how frequently they assess lungs on CT, how eager they are to try new information technologies, and how frequently they used computer-aided detection tools), these factors were included in the multilevel regression analyses as well. The same analysis scheme was used for all multilevel logistic regression analyses. First, an empty model was run to identify the variance at the individual level. The second regression analysis also considered the variants of onboarding tutorials and AI output. Third, whether the same nodules were found by AI and the radiologist exclusively and whether they made changes in the number of reported nodules were added. The final analysis also included different CT scans and radiologists’ characteristics.

A *P* value of <.05 was considered statistically significant. All analyses were performed using Stata (version 17; StataCorp).

### Ethical Considerations

This study was approved by the Internal Committee for Biomedical Experiments of Philips (number ICBE-S-000204) and conducted in accordance with the Declaration of Helsinki (as revised in 2013). Written informed consent was obtained from the participating clinicians.

## Results

### Participants

In total, 20 physicians from 7 Dutch hospitals participated in this study. Of the 20 participants, 16 were radiologists (median 10.5, range 1-32 years of experience as a specialist), 1 was a nuclear radiologist (2 years of experience in assessing lung CT scans), and 3 were radiology residents (median 2, range 1-5 years of residency). Of the 16 radiologists, 8 (50%) specialized in thoracic radiology. The male-to-female ratio was 50:50. Of the participants, 25% (5/20) were aged between 26 and 35 years, 35% (7/20) were aged between 36 and 45 years, 20% (4/20) were aged between 46 and 55 years, and 20% (4/20) were aged between 56 and 65 years.

### Mental Model and Psychological Trust

**Figure 5:**
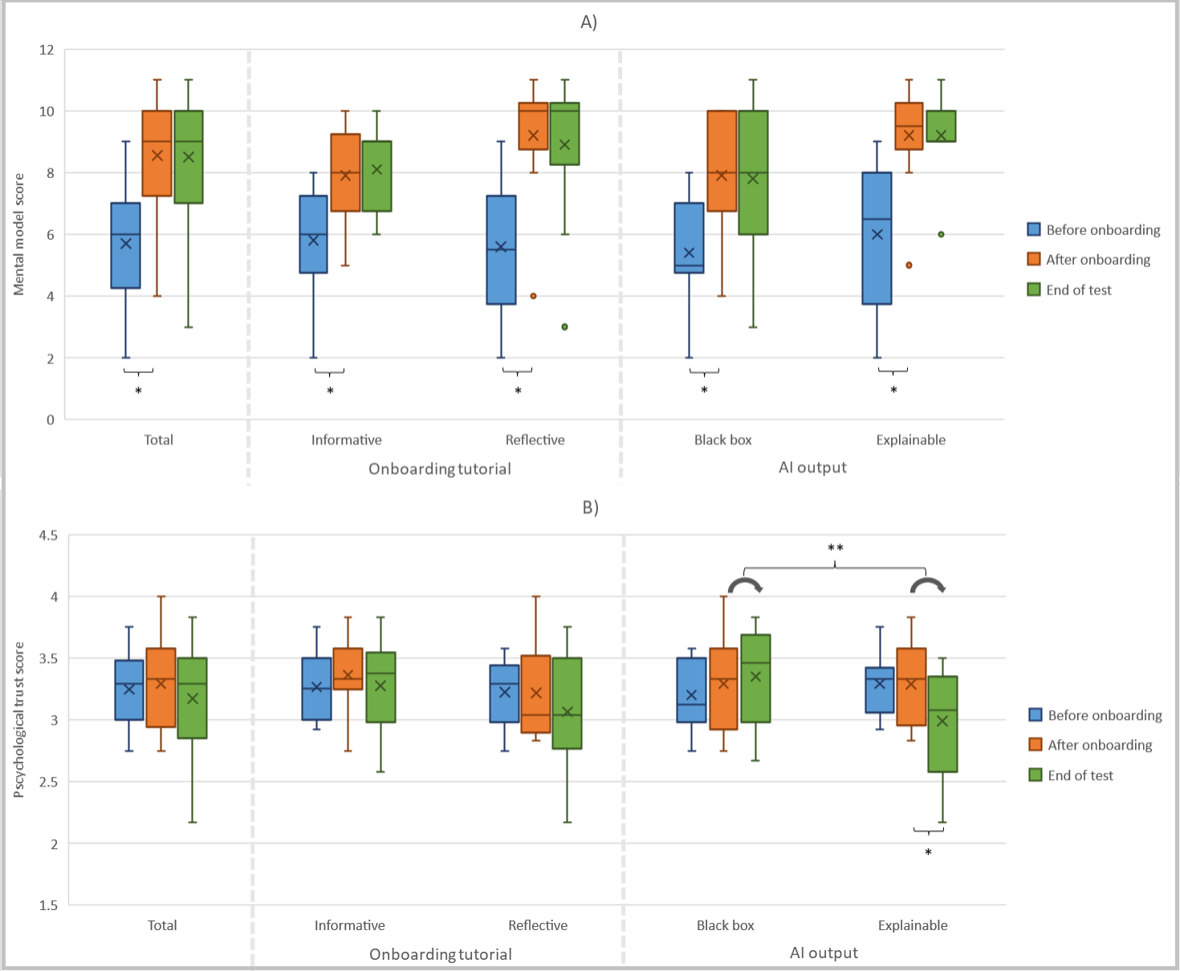
Boxplot showing the (A) mental model scores and (B) psychological trust scores before and after onboarding and at the end of the test using either informative or reflective onboarding tutorials and either black box or explainable artificial intelligence (AI) output. The cross shows the mean value; the horizontal line inside the box indicates the median value; the lower and higher boundaries of the box indicate the first and third quartiles; the whiskers indicate the minimum and maximum values; and outliers are indicated by colored dots. Only significant differences are mentioned. *Significant difference between time points. **Significant difference in the change over time between the black box and explainable AI output groups.

Figure 5 presents the mental model and psychological trust scores before onboarding, after onboarding, and at the end of the test. These scores were shown for all radiologists together and for the 2 variations of the onboarding tutorials and AI output separately.

After onboarding, the mental model score of the radiologists was significantly higher than that before onboarding (*P*<.001). The mean scores were 5.7 (SD 2.0) before onboarding and 8.6 (SD 1.9) after onboarding, which supports hypothesis 3a. Both informative (*P*=.01) and reflective (*P*=.01) onboarding resulted in significantly higher mental model scores. These improvements did not significantly differ between the groups; therefore, hypothesis 4a is not supported. At the end of the test, the mental model scores did not differ significantly from the scores after onboarding in any of the groups, which does not support hypothesis 1a and hypothesis 6a.

Considering all radiologists together, the psychological trust scores did not change significantly over time; therefore, hypotheses 1b and 3b are not supported. Between the 2 variations of onboarding tutorials, no significant differences in psychological trust scores were observed, and therefore, hypothesis 4b is not supported. In the group that received explainable AI output, psychological trust at the end of the test was significantly lower than that after onboarding (*P*=.02), which interestingly contradicts hypothesis 6b. In the group that received black box AI output, there was no significant change in psychological trust. Changes in psychological trust scores between after onboarding and at the end of the test were significantly different between the black box output and explainable AI output groups (*P*=.03). All *P* values can be found in Multimedia Appendix 5.

None of the tested characteristics of radiologists significantly predicted the mental model scores or the psychological trust scores at the different time points nor did they significantly predict the changes over time.

### Use of AI Recommendations

**Figure 6:**
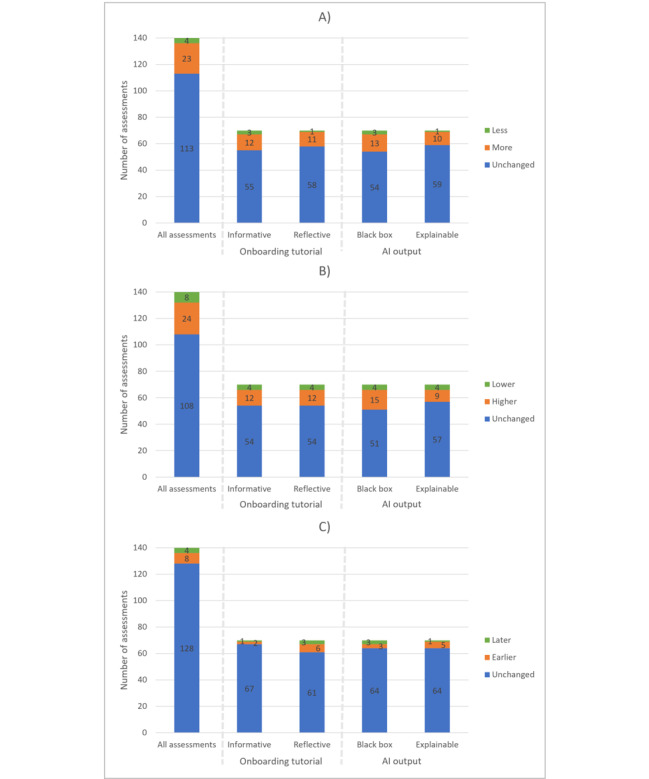
Bar graph showing the changes in the radiologist’s computed tomography assessments; (A) Reported nodules, (B) Malignancy probability, (C) Follow-up advice after viewing the recommendations from the artificial intelligence–based computer-aided detection or diagnosis using either informative or reflective onboarding tutorials, and either black box or explainable artificial intelligence (AI) output. No significant differences between the onboarding and AI output groups resulted from the multilevel regression analyses.

After viewing the AI outcomes, the radiologists adjusted their found nodules in 27 of 140 assessments, their estimated probability of malignancy in 32 of 140 assessments, and their follow-up advice in 12 of 140 assessments (Figure 6). Radiologists predominantly added nodules (23 of 27 changed cases), increased the probability of malignancy (24 of 32 changed cases), and shortened the recommended follow-up period (eg, from CT at 6-12 months to CT at 3-6 months; 8 of 12 changed cases). The empty model, which included no predictor variables, revealed that regarding whether radiologists made changes, approximately 3% of the variance in the outcome variable was attributable to differences between radiologists. For changes in malignancy prediction and follow-up advice, this attributable variance was approximately 20% and 7%, respectively. This indicates that there is some variability in the outcome, which can be explained by the individual radiologists. Radiologists’ assessments were not significantly impacted by the type of onboarding tutorial or by the type of AI output; therefore, hypotheses 5 and 7 are not supported. All outcomes of the multilevel regression analyses can be found in Multimedia Appendix 6.

**Figure 7:**
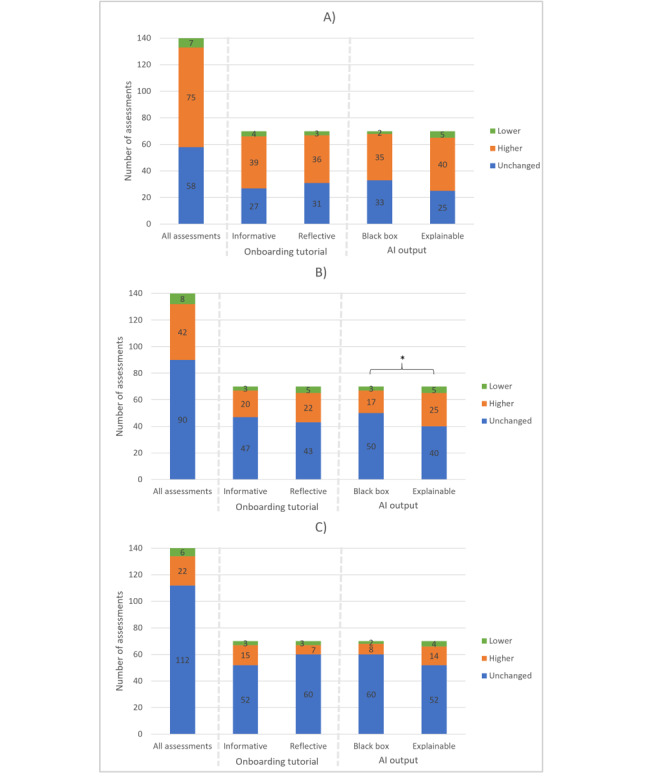
Bar graph showing the changes in the radiologist’ confidence in their assessments; (A) Confidence reported nodules, (B) Confidence malignancy probability, (C) Confidence follow-up advice after viewing the recommendations from the artificial intelligence–based computer-aided detection or diagnosis using either informative or reflective onboarding tutorials, and either black box or explainable artificial intelligence (AI) output. *The multilevel regression analysis showed a significant difference between the 2 groups according to the number of changed radiologists’ confidence (orange+green) in their assessment after using the artificial intelligence–based computer-aided detection or diagnosis system.

At all levels of assessment, radiologists’ confidence in the assessments (n=140) predominantly increased after viewing the AI-CAD recommendations (in found nodules [75/82, 91%] of all changed assessments, in malignancy probability [42/50, 84%], in follow-up advice [22/28, 79%]; Figure 7), which supports hypothesis 2. The multilevel regression analysis revealed that in the empty model without predictor variables, approximately 20% of the total variance in the changed confidence in detected nodules was attributed to differences between radiologists. Regarding the changed confidence in malignancy prediction and follow-up advice, this attribution of the total variance was 10% and 7%, respectively. The radiologists’ confidence in their assessments was not significantly affected by the type of onboarding tutorial but was affected by the type of AI output after controlling for whether the AI model found the same or different nodules as the radiologist without AI assistance (first model: β=0.143; *P*=.16; second model: β=0.167; *P*=.04; third model: β=0.207; *P*=.02). See Multimedia Appendix 6 for all outcomes of the multilevel regression analyses.

### Secondary Outcomes

#### Post Hoc Analyses Regarding the Use of AI Recommendations

In 26 of 140 assessments, the same nodules exclusively had been found by the AI model and the unassisted radiologist. In these cases, radiologists changed the number of nodules less frequently than when different nodules had been found (second model: β=–0.245; *P*=.003 third model: β=–0.437; *P*=.001; Multimedia Appendix 6).

In 27 of 140 assessments, radiologists changed the number of nodules when using AI assistance. In the cases in which the radiologists did not change the number of nodules, the radiologists’ confidence in their malignancy prediction changed more often, mostly increased, than in the cases in which the radiologists did change the number of found nodules (second model: β=0.369; *P*<.001; third model: β=0.283; *P*=.001; Multimedia Appendix 6). Whether the number of nodules was changed also significantly influenced radiologists’ confidence in their follow-up advice, but this was probably related to some radiologists’ characteristics, as this effect disappeared after controlling for such characteristics (second model: β=0.277; *P*=.02; third model: β=0.154; *P*=.23).

#### Correctness Follow-Up Advice

Without AI assistance, the radiologists provided the correct follow-up advice according to the Fleischner criteria in 94 of 140 assessments (Table 1). Mostly, the correct follow-up advice was provided for CT cases 1, 3, 5, and 7, whereas most of the incorrect follow-up advice concerned CT cases 2, 4, and 6. With AI assistance, radiologists provided correct follow-up advice in 100 of 140 assessments. In 12 cases, the follow-up advice was changed after viewing the AI results. In 7 of these 12 cases, correct follow-up was provided after seeing the AI results. In 1 case, correct follow-up advice that was given initially was changed to incorrect follow-up advice after seeing the AI results. In 3 cases, the changed follow-up advice was still not correct but closer to the correct follow-up advice, and in the remaining case, the changed follow-up advice was further from the correct follow-up advice.

**Table 1:** Correct follow-up advice provided by the radiologists.

CT^a^ cases (number of assessments)		All (n=140)	CT1 (n=20)	CT2 (n=20)	CT3 (n=20)	CT4 (n=20)	CT5 (n=20)	CT6 (n=20)	CT7 (n=20)
Correct follow-up advice given without AI^b^ assistance, n (%)		94 (67)	20 (100)	6 (30)	17 (85)	6 (30)	20 (100)	7 (35)	18 (90)
Correct follow-up advice given with AI assistance, n (%)		100 (71)	20 (100)	7 (35)	17 (85)	7 (35)	20 (100)	9 (45)	20 (100)
**Changed follow-up advice after using AI assistance, n (%)**		12 (9)	0 (0)	2 (10)	1 (5)	5 (25)	0 (0)	2 (10)	2 (10)
	Wrong→correct	7 (58)	0 (0)	1 (50)	0 (0)	2 (40)	0 (0)	2 (100)	2 (100)
	Wrong→better (still wrong, but closer to correct follow-up)	3 (25)	0 (0)	1 (50)	1 (100)	1 (20)	0 (0)	0 (0)	0 (0)
	Wrong→worse (still wrong, even further from correct follow-up)	1 (8)	0 (0)	0 (0)	0 (0)	1 (20)	0 (0)	0 (0)	0 (0)
	Correct→wrong	1 (8)	0 (0)	0 (0)	0 (0)	1 (20)	0 (0)	0 (0)	0 (0)

^a^CT: computed tomography.

^b^AI: artificial intelligence.

## Discussion

### Principal Findings

Our study demonstrated that onboarding is of great importance because the radiologists’ mental model of the AI-CAD system was significantly more accurate after onboarding. This finding implies that after onboarding, radiologists had a better understanding of the capabilities and limitations of the AI-CAD system, which is important for using the AI recommendations correctly. In addition, the importance of onboarding was emphasized by the fact that the mental model did not become more accurate through the actual use of the AI-CAD system. A study by Lam Shin Cheung et al [45] supports the need for onboarding.

We hypothesized that reflective onboarding would result in a more appropriate level of trust than informative onboarding, as radiologists in the reflective onboarding group were triggered to actively engage in cognitive reflection and receive feedback on their mental model. However, this hypothesis was not supported because the increases in mental model scores of radiologists in the reflective onboarding group did not significantly differ from those in the informative onboarding group. This unexpected finding might be explained by the high level of clarity of the explanations provided during both informative and reflective onboarding, because of which the reflection had no significant added value. Alternatively, participating radiologists might possess a natural tendency to engage in cognitive reflection even if the system does not actively trigger them to do so.

Another unexpected finding was that explainable AI output resulted in a significant decrease in psychological trust (*P*=.02) during the use of the AI-CAD system for assessing the 7 CT scans, which was not the case in the group that received black box AI output (Figure 5). Apparently, users can become insecure about the reliability of AI-CAD when they receive explanations. On the basis of feedback from the participating radiologists, we know that some radiologists observed that the AI-CAD system provided different malignancy predictions for similar nodules with the same visual characteristics provided such as size and morphology. These discrepancies raised questions about why nodules with similar characteristics had different malignancy probabilities. In fact, this key aspect still felt like a black box to the participants. Apparently, providing more transparency, which enables radiologists to observe inconsistencies in the AI predictions, can decrease the radiologists’ trust in the AI-CAD system. However, this decrease in trust might be appropriate because the AI model’s performance might be suboptimal and inconsistent.

In many CT assessments, the radiologists did not make any changes in their assessments after seeing the AI recommendations. However, this does not necessarily mean that the radiologist did not trust the AI-CAD system. There can be several reasons for making no changes. First, the AI recommendations can be exactly the same as the radiologists’ assessments. Second, radiologists may disagree with the AI recommendations, which may be appropriate because the AI model also makes mistakes. Third, concerning malignancy prediction and follow-up advice, the AI recommendations may not impact the assessments, whereas the radiologists do agree with the AI recommendations. For instance, the AI model might find an extra nodule; however, if another larger and more suspicious nodule was already detected, the extra nodule does not impact the radiologist’s malignancy risk prediction at the patient level or the follow-up recommendation.

Another important finding is that radiologists became more confident in their assessments after using the AI recommendations. This change might be explained by the fact that the AI-CAD system provides an extra check, which reduces the likelihood of nodules being overlooked. Hence, it provides radiologists with a sense of safety that increases their confidence, regardless of whether they agree with the AI output.

The follow-up advice was adjusted by the radiologists after viewing the AI results in only 12 of 140 assessments, whereas the number of observed nodules and the malignancy probabilities were changed more often (27/140, 19.3% assessments and 32/140, 22.9% assessments, respectively). This finding can be explained by the fact that follow-up advice is predominantly affected by the most suspicious nodule. Consequently, an AI-CAD finding of an additional small nodule while a large suspicious nodule had already been detected by the radiologist did not impact the radiologist’s follow-up advice. Of the 3 assessment levels, follow-up advice is clinically most relevant. When the follow-up advice was adjusted, it was mostly changed to a shorter follow-up period (8/12, 67% assessments; eg, from CT at 6-12 months to CT at 3-6 months). This finding indicates that, owing to the AI recommendations, radiologists tended to be more careful and took fewer risks in their follow-up advice. For this study, earlier follow-up was appropriate as all CT scans showed cancer cases, but in clinical practice, it can be questionable whether being more careful and taking fewer risks in the follow-up advice is always desirable because it may increase the health care costs. Therefore, it is of great importance to study the cost-effectiveness of AI-CAD systems.

### Secondary Findings

Confidence in malignancy prediction was significantly more frequently changed when the radiologist did not change their number of nodules after viewing the AI recommendations (Multimedia Appendix 6). This might be caused by the malignancy prediction provided by the AI-CAD system of nodules that they also found themselves. The radiologist might become more convinced whether a case is malignant or benign based on this AI-CAD malignancy recommendation.

This study also demonstrates the importance of applying a user-centered design process to achieve appropriate use of the AI-CAD system. This is lacking in many studies and applications [46]. Radiologists indicated in their feedback that the PPV and NPV were difficult to interpret. Therefore, different visualizations of model confidence might be more appropriate, such as using only bar graphs. Furthermore, radiologists mentioned that some extra functionalities that radiologists use in clinical practice for lung assessment need to be implemented in the prototype, such as multiplanar reconstruction and maximum intensity projection, underlining the need for tight integration of AI into the radiologist routine workstations. In addition, they mentioned that during onboarding, they would like to receive more information on AI model training and validation, including the data sets used and ground truth definition, which should therefore be added to the onboarding prototype. This need is in line with the findings of Cai et al [31], who explored the information needs for onboarding for AI-CAD in pathology. Ashoori and Weisz [43] mentioned that information on AI model training and testing is important for radiologists’ trust in AI-CAD systems. Radiologists’ feedback needs to be incorporated to achieve the AI-CAD system that fully meets radiologists’ needs.

### Limitations and Future Perspectives

This study had several limitations. First, this study was not fully representative of the clinical situation. Owing to time constraints, we specifically asked the radiologists not to assess the entire case but to focus on the component task of lung nodule assessment. Therefore, radiologists were aware that lung nodule assessment was important, which is representative for CT scans acquired because of pulmonary complaints but not for scans with incidental lung nodules. In addition, this study exclusively included scans of cancer cases, which differs from clinical practice, in which scans may also show no nodules and solely benign nodules. However, the data set with cancer cases was appropriate for our research goals.

Second, in the current prototype, the explainable AI output was simulated post hoc. There is an increasingly louder call to build causal models in the medical domain where the cost of failure is high, allowing the clinician to verify the causal chain of effects of clinically validated features on the model prediction. However, such inherently interpretable models are currently the exception rather than mainstream practice [47]. In this study, we focused on the current state of medical practice, where, if at all, most post hoc explainability techniques are used to improve interpretability. Importantly, post hoc techniques come at the expense of the validity of the relationship between post hoc explanations and model prediction. In fact, what appears to an end user as an explanation might not convey why the black box predicted what it did [48]. In this study, we were interested in the effect of a widespread approach to explain user trust and decision-making in a medical context. In addition, although simulating explainable AI output is very useful in the early stages of AI-CAD system development [33,34], having fully functioning AI models would further add to the realism of the test. Furthermore, it would be valuable if the algorithm can provide the extent to which each nodule characteristic contributed to malignancy prediction. In addition, PPV and NPV computed at the patient level were applied at the nodule level.

Third, this study included only 20 radiologists and 7 CT scans, which need to be scaled up to have sufficient power to be able to detect smaller effect sizes. In this pilot study, this limitation was accepted to make the test less time-consuming for the participating radiologists and to postpone larger samples after at least some evidence of larger effects in this context could be established. During case selection for this study, we aimed to collect a mix of relatively easy and more challenging cases, which worked well, considering the number of correct follow-up recommendations in Table 1. In a future large-scale study, it would be advisable to use a clinically representative data set to prevent the impact of selection bias. Testing on a larger scale is also required to analyze what radiologists do with FP findings and how these findings affect their trust in the AI-CAD. It is interesting to assess which types of FP findings are recognized by radiologists. Furthermore, it is useful to analyze whether changes in the number of observed nodules and in malignancy probability are correct based on a reference standard defined by expert radiologists and pathology. This is important because of automation bias, implying that radiologists rely too much on the AI recommendations, has to be prevented [40,49].

### Conclusions

When clinical decision support systems are implemented, clinicians should receive careful onboarding that gives them a better understanding of the capabilities and limitations of the AI-CAD system. This understanding contributes to appropriate trust in the AI system, which is important when AI systems are used in clinical practice. Providing more AI output transparency, which enables clinicians to observe inconsistencies in the AI recommendations, can decrease clinicians’ trust in the AI-CAD system. AI recommendations frequently increased radiologists’ confidence in their assessments, even if they did not fully agree with these recommendations.

## Appendix

Multimedia Appendix 1 Characteristics of computed tomography cases.

Multimedia Appendix 2 Positive predictive value and negative predictive value definitions.

Multimedia Appendix 3 Experimental conditions.

Multimedia Appendix 4 Forms for measuring trust.

Multimedia Appendix 5 Mental model and psychological trust.

Multimedia Appendix 6 Use of artificial intelligence recommendations.

## Abbreviations

AI: artificial intelligence
AI-CAD: artificial intelligence–based computer-aided detection or diagnosis
CT: computed tomography
FP: false-positive
NPV: negative predictive value
PPV: positive predictive value
