# Supplementary material for: The Impact of Expectation Management and Model Transparency on Radiologists’ Trust and Utilization of AI Recommendations for Lung Nodule Assessment on Computed Tomography: Simulated Use Study
Source: JMIR AI. 2024 Mar 13;3:e52211. doi: 10.2196/52211 (PMC11041414; doi:10.2196/52211)
Supplement: Multimedia Appendix 1 [file ai_v3i1e52211_app1.docx]

# Appendix 1 – Characteristics CT cases

*Table 1.1: Characteristics of the lung nodules of the seven CT cases.*

| **Case** | **Lung nodules found by AI** | **Further description on case** | **Provided follow-up** | **Final diagnosis** |
| --- | --- | --- | --- | --- |
| **CT1** | N = 3   - 2 TP   - Solid, spiculated, 21 mm   - Ground glass, smooth, 15 mm - 1 FP   - Vocal cord | - The solid, spiculated nodule is clearly highly suspicious | - PET-CT - Bronchoscopy and cytology - VATS and lobectomy | Non-small cell lung carcinoma (squamous cell carcinoma) |
| **CT2** | N = 5   - 5 TP   - Solid, spiculated, 9 mm   - 4 Ground glass, smooth, 8 mm, 11 mm, 9 mm and 9 mm | - The solid spiculated nodule is located against veins - Several small nodules (<6 mm) visible | No follow-up  Retrospectively*: CT at 3 months, PET/CT or biopsy | Pathology not acquired because of bad pulmonary function, but progression on CT scan and FDG uptake on PET-CT, therefore expected to be malignant |
| **CT3** | N = 2   - 2 TP   - Solid, spiculated, 15 mm   - Subsolid, lobulated, 7 mm | - The solid, spiculated nodule is clearly highly suspicious, located high in the right upper lobe - Emphysema, fibrosis, atelectasis | No follow-up  Retrospectively*: CT in 3 months, PET/CT or biopsy | Non-small cell lung carcinoma (adenocarcinoma) |
| **CT4** | N = 2   - 1 TP   - Solid, smooth, 7 mm - 1 FP   - Pulmonary artery | - The solid, smooth nodule has subpleural location | No follow-up  Retrospectively*: CT in 6-12 months | Pathology shows no malignant cells, but progression on CT scan and FDG uptake on PET-CT, therefore still expected to be malignant |
| **CT5** | N = 5   - 1 TP   - Solid, spiculated, 13 mm - 4 FP   - 2 fibrotic strands   - Pericardial fat pad   - Residual abnormality | - The solid, spiculated nodule is clearly highly suspicious | - PET-CT - Bronchoscopy and cytology - VATS and lobectomy | Non-small cell lung carcinoma (adenocarcinoma) |
| **CT6** | N = 2   - 1 TP   - Ground glass, smooth, 15 mm - 1 FP   - Pericardial fat pad | - The ground glass, smooth nodule has subpleural location | No follow-up  Retrospectively*: CT in 6-12 months | Non-small cell lung carcinoma (adenocarcinoma) |
| **CT7** | N = 1   - 1 TP   - Solid, spiculated, 10 mm | - The solid, spiculated nodule is clearly highly suspicious, located high in the right upper lobe - Motion artifacts, caused by breathing | - PET-CT - Bronchoscopy and cytology - CT thorax | Pathology not acquired, but progression on CT scan and FDG uptake on PET-CT, therefore expected to be malignant |

*Abbreviations: FP, False Positive; TP, True Positive; FDG, fluorodeoxyglucose; PET-CT, Positron Emission Tomography–Computed Tomography; VATS, Video-assisted thoracic surgery*

**Retrospectively implies that the correct follow-up according to the Fleischner criteria was retrospectively determined by two expert radiologists in consensus.*
