# Supplementary material for: The Impact of Expectation Management and Model Transparency on Radiologists’ Trust and Utilization of AI Recommendations for Lung Nodule Assessment on Computed Tomography: Simulated Use Study
Source: JMIR AI. 2024 Mar 13;3:e52211. doi: 10.2196/52211 (PMC11041414; doi:10.2196/52211)
Supplement: Multimedia Appendix 2 [file ai_v3i1e52211_app2.docx]

# Appendix 2 – PPV and NPV definitions

This section describes the approach for generating the model predictions at different confidence levels. The framework described in the study of *Trajanovski et al*. have been employed [35]. This model was trained using the National Lung Screening Trial (NLST) dataset and can produce a lung cancer probability for a CT scan. The neural network used in this paper computes the CT scan malignancy probability using a max-pooling layer to aggregate the nodule-level malignancy probabilities, thus the individual nodule malignancy probability can also be extracted. The NLST dataset is a lung cancer screening dataset, that contains low-dose CT scans and the patient distribution is based on the lung cancer screening criteria.

This model was, without retraining, applied to a dataset of 200 scans from the Dutch top clinical hospital. This dataset was not related to lung cancer screening and contained normal-dose CT scans. The model produced a malignancy probability for both the full CT scans as well as a malignancy probabilities for the identified nodules. In order to compute the probability intervals that correspond to different levels of confidence for the model, entropy measure was used that is a common baseline method in the research literature [38]. Binary entropy simply assigns values close to 0.5 as having high uncertainty and values close to 0 and 1 as having low uncertainty. Since the hospital dataset was highly imbalanced, binary cross-entropy was used to quantify model confidence.

Since scan parameters of the scans of the hospital as well as the patient distribution were different compared to the model’s training data, temperature scaling was used to calibrate the model’s output probabilities [39]. In order to calibrate the output probabilities for the hospital dataset and compute the performances at the different confidence levels, the following process was followed:

- 2000 training/validation data splits were created for the hospital data.
- Temperature scaling was employed to calibrate the output probabilities using the training part of the split.
- Temperature scaling was applied on the validation part of the split.
- Using cross-entropy as an uncertainty measure, on the validation split the probability intervals were computed that correspond to the top-10% confidence predictions, top-20% confidence predictions, and so on.
- For each confidence group, performance statistics were computed (Negative predictive values (NPV) and positive predictive values (PPV)).

Based on the process described above, the mean NPV and PPV performances were computed, as well as the mean probability intervals for each confidence group. These statistics were computed based on scan level performance measurements, since the labels (cancer diagnosed, no cancer) were only available at patient level. In the employed tool, these statistics were displayed at nodule level, which can be interpreted as the potential NPV/PPV scores if this is the only nodule that is contained in the scan.
