# Supplementary material for: The Impact of Expectation Management and Model Transparency on Radiologists’ Trust and Utilization of AI Recommendations for Lung Nodule Assessment on Computed Tomography: Simulated Use Study
Source: JMIR AI. 2024 Mar 13;3:e52211. doi: 10.2196/52211 (PMC11041414; doi:10.2196/52211)
Supplement: Multimedia Appendix 3 [file ai_v3i1e52211_app3.docx]

# Appendix 3 – Experimental conditions

## 3.1. AI recommendations

*Table 3.1.1: Overview of the information cues of the AI recommendations.*

| **AI recommendations (per nodule)** | **Values** | **Actual or simulated AI output** | **Included in black box AI output** | **Included in explainable AI output** |
| --- | --- | --- | --- | --- |
| **Nodule detection and localization** | - Location of nodule centerpoint in CT slice stack | Actual | Yes | Yes |
| **Malignancy classification** | - Benign or malignant | Actual | Yes | Yes |
| **Malignancy classification confidence** | - Summarized by graph bar (low-medium-high) - PPV or NPV | Actual | Yes | Yes |
| **Nodule characteristics**  **(i.e., rationale for malignancy classification)** | - Long axis (mm) - Margin (smooth, spiculated) - Solidity (solid, sub-solid, ground-glass) - Location (lung lobe) | Simulated | No | Yes |

*Figure 3.1.1: Example of AI finding by black box AI. Figure 3.1.2: Example of AI finding by explainable AI.*

## 3.2. Onboarding tutorials

Scans were shown in the user interface. Linked to the associated parts of the scans and interface, information was interactively provided to the radiologists by pop-up messages. The following information was provided: the tasks executed by the algorithm (nodule detection and malignancy prediction), that the malignancy prediction was only based on the local area around the nodule (and so does not take into account patient context) and common pitfalls of the algorithm (see all provided pop-up messages in Table B2.1). In case of reflective onboarding, the radiologists additionally received four questions and the system confirmed their expectation in case their expectations did match the AI capabilities or provided an additional explanation in case their expectations did not match the AI capabilities (see all questions in Table B2.2).

*Table 3.2.1: Explanations provided in the (informative and reflective) onboarding tutorials.*

| **Topic** | **Information** |
| --- | --- |
| Introduction | Welcome to the introduction of this AI model. The most important aspects of the AI-CAD will be explained to you in a stepwise way. You can use the previous and next buttons to navigate through this introduction. Click start to start. |
| Lung nodule detection | This AI model is applied to diagnostic chest CT images and performs two tasks. First, it detects nodules/lesions in the entire lungs. |
| Malignancy prediction | Second, it provides a malignancy prediction for each individual lung nodule/lesion. |
|  | For the malignancy prediction, the AI model only analyses a cube of 28 mm around the center of the detected lung nodule/lesion. |
|  | It predicts whether the nodule is more likely to be malignant or benign. |
|  | For this malignancy prediction, the model takes into account nodule features such as nodule size (long axis), margin, solidity and location. |
| Confidence malignancy prediction | These nodule features influence the confidence of the prediction (malignant or benign). The more bars that are filled white, the higher the confidence. |
|  | The malignancy prediction can be seen as a test for malignancy. Therefore, the confidence for a malignant prediction comes with a positive predictive value (PPV). The PPV is the probability that a nodule with a malignant prediction is truly malignant, based on pathology. |
|  | The confidence bars of the **malignant** predictions are based on the following PPV values: 1 bar filled PPV = 0.25, 2 bars filled PPV = 0.30, 3 bars filled PPV = 0.38. |
|  | The confidence for a benign prediction comes with a negative predictive value (NPV). The NPV is the probability that a nodule with a benign prediction is truly benign, based on pathology. |
|  | The confidence bars of the **benign** predictions are based on the following NPV values: 1 bar filled NPV = 0.94, 2 bars filled NPV = 0.97, 3 bars filled NPV > 0.99. |
|  | As an example, this AI finding means: this nodule is malignant with a high confidence, because it is large, spiculated and solid. |
| Limitations AI model | Although this AI model has comparable performance to radiologists in estimating the malignancy probability, the model is no human and operates differently. |
|  | There are a few types of errors that this AI model typically tends to make due to how it has been trained. |
|  | First of all, it might identify a nodule/lesion while it is actually another (normal) structure, e.g., a pericardial fat pad. |
|  | Secondly, it might identify a nodule as malignant, while it is actually another type of abnormal lung tissue, e.g., pulmonary fibrosis. This can happen because the training of the AI model was not focused on discriminating between different types of abnormalities in the lungs. |
|  | As a final note, keep in mind that the AI is meant to be a second reader to support your clinical assessment. |

*Table 3.2.2: Additional questions and explanations of the reflective onboarding tutorial.*

| **Additional questions reflective onboarding** |
| --- |
| Now that you are familiar with this AI system, you will be shown a number of practice cases that are based on real (retrospective) patient cases. You will also receive feedback on your understanding of the system.  During this practical case, the system is not interactive. Only the slice where the AI found something suspicious will be shown to you. Be sure to review the AI findings before clicking next. |
| This is the first practical case.  Do you think this nodule/lesion is suspicious? Do you agree with the AI prediction? |
| - Do you think the AI model took into account secondary signs of lung cancer (e.g., atelectasis) for the prediction of the cancer probability?   Yes: Keep in mind that the AI model does not take secondary signs of lung cancer into account. It only analyzes at a cube of 28 mm around each detected nodule/lesion.  No: That’s right, secondary signs of lung cancer are not taken into account by the AI model. It only analyzes at a cube of 28 mm around each detected nodule/lesion. |
| - Did the AI make you confident that there are no loco-regional and distant metastases?   Yes: Be mindful that loco-regional and distant metastases are not analyzed by the AI model. It only takes the CT volume of the lungs as input, not the full chest.  No: Indeed, loco-regional and distant metastases are not analyzed by the AI model. It only takes the CT volume of the lungs as input, not of the full chest. |
| This is the second practical case.  Here you can see that the AI model completely missed a malignant lung nodule. |
| So make sure to always check the complete CT scan yourself. |
| - Do you expect that for the malignancy prediction, the AI model explicitly considers characterized nodule features, such as nodule size, margin, solidity and location?   Yes: Indeed, the characteristics of the detected nodule influence the malignant/benign prediction and the confidence of the prediction.  No: Be aware that the characteristics of the detected nodule do influence the malignant/benign prediction and the confidence of the prediction. |
| This is the final practical case. In this case, two AI findings will be shown to you. |
| This is the first AI finding.  Do you think this nodule/lesion is suspicious? Do you agree with the AI model’s prediction? |
| This nodule/lesion was later confirmed to be cancerous. |
| This is the second AI finding.  Do you think this nodule/lesion is suspicious? Do you agree with the AI model’s prediction? |
| This case is an example of a typical AI model mistake. The AI model has identified a nodule as malignant, although it is actually another type of abnormal lung tissue, namely benign pleural thickening. |
| - Do you think this AI model combined the information from both detected nodules into the malignancy predictions?   Yes: Please note that the AI model does not combine information from multiple nodules into the malignancy predictions.  No: Exactly, the AI model does not combine information from multiple nodules into the malignancy predictions. |
| This is the end of the interactive introduction. Click finish to close it. |
