# Supplementary material for: The Impact of Expectation Management and Model Transparency on Radiologists’ Trust and Utilization of AI Recommendations for Lung Nodule Assessment on Computed Tomography: Simulated Use Study
Source: JMIR AI. 2024 Mar 13;3:e52211. doi: 10.2196/52211 (PMC11041414; doi:10.2196/52211)
Supplement: Multimedia Appendix 4 [file ai_v3i1e52211_app4.docx]

# Appendix 4 – Forms for measuring trust

## 4.1. Mental model

Detecting nodules/lesions

The following questions are related to your expectations of the detection of nodules/lesions by the AI model.

|  | **Questions** | **Answers** | | |
| --- | --- | --- | --- | --- |
| 1 | I expect that this AI model can detect lung nodules/lesions. | Yes | No | I don’t know |
| 2 | I expect that this AI model can detect loco-regional and distant metastases. | Yes | No | I don’t know |
| 3 | I expect that for detecting nodules/lesions, this AI model takes the full CT volume *of* *the* *lungs* as input. | Yes | No | I don’t know |
| 4 | I expect that for detecting nodules/lesions, this AI model takes the full CT volume as input, in addition to the CT volume of the lungs. | Yes | No | I don’t know |
| 5 | I expect that this AI model can discriminate between lung cancer and other abnormalities, such as infectious granuloma. | Yes | No | I don’t know |

Malignancy prediction

The following questions are related to your expectations of the malignancy prediction that is provided by the AI model.

|  | **Questions** | **Answers** | | |
| --- | --- | --- | --- | --- |
|  | I expect that for the malignancy prediction, this AI model considers… |  | | |
| 6 | … the detected nodule and its direct surroundings. | Yes | No | I don’t know |
| 7 | … secondary signs of lung cancer, such as involvement of main  bronchus, atelectasis, or invasion of the pleura. | Yes | No | I don’t know |
| 8 | … other contributing imaging features, such as other pulmonary  disease, e.g., emphysema. | Yes | No | I don’t know |
| 9 | … patient factors, such as age, smoking history, gender, etc. | Yes | No | I don’t know |
| 10 | … information from prior scans, if available. | Yes | No | I don’t know |
| 11 | If this AI model detects multiple suspicious nodules in one CT scan, I expect that this AI combines the information from these nodules into the malignancy prediction. | Yes | No | I don’t know |

## 4.2. Psychological trust

|  | **Questions** | **Strongly**  **disagree** | **Disagree** | **Neutral** | **Agree** | **Strongly**  **agree** |
| --- | --- | --- | --- | --- | --- | --- |
| 1 | This AI model is trustworthy. | 1 | 2 | 3 | 4 | 5 |
| 2 | I would change one or more aspects of this  AI model to make it trustworthy.* | 1 | 2 | 3 | 4 | 5 |
| 3 | This AI model will produce a fair outcome  for the patient affected by the decision. | 1 | 2 | 3 | 4 | 5 |
| 4 | I need more information about how the AI model was trained and tested in order to  trust the AI model.* | 1 | 2 | 3 | 4 | 5 |
| 5 | This AI model would always make the same recommendation under the same  conditions. | 1 | 2 | 3 | 4 | 5 |
| 6 | The AI model output will be consistent with  decisions made for similar patient cases. | 1 | 2 | 3 | 4 | 5 |
| 7 | The use of this AI model is appropriate for detecting and interpreting incidental lung  nodules. | 1 | 2 | 3 | 4 | 5 |
| 8 | The AI model output is based on reliable  information. | 1 | 2 | 3 | 4 | 5 |
| 9 | I trust that the technical implementation of  the AI model is correct. | 1 | 2 | 3 | 4 | 5 |
| 10 | I am confident in this AI model. I feel that it  works well. | 1 | 2 | 3 | 4 | 5 |
| 11 | I am wary of this AI model.* | 1 | 2 | 3 | 4 | 5 |
| 12 | I like this AI model. | 1 | 2 | 3 | 4 | 5 |

** For questions 2, 4 and 11, the scores were reversed.*

## 4.3. Utilization of AI recommendations

|  | **Questions** **and** **answers** | | | | | **Please** **rate** **your** **confidence** **in** **the** **provided**  **answer** **on** **the** **left** | | | | |
| --- | --- | --- | --- | --- | --- | --- | --- | --- | --- | --- |
| 1 | How many lung nodules/lesions have you found in this chest CT?  nodules/lesions | | | | | **1**  Not confident  at all | **2** | **3** | **4** | **5**  Very confident |
| 2 | Please estimate the malignancy probability for this chest CT (at patient level, between 0% and 100%).  % | | | | | **1**  Not confident  at all | **2** | **3** | **4** | **5**  Very confident |
| 3 | Which follow-up would you choose for this patient  (according to the Fleischner criteria)? | | | | | **Please** **rate** **your** **confidence** **in** **the** **provided**  **answer** **on** **the** **left** | | | | |
|  | **1**  Consider CT in 3 months, PET/CT,  or tissue sampling | **2**  CT in 3-6  months | **3**  CT in 6-12  months | **4**  CT in 12  months | **5**  No routine  follow-up | **1**  Not confident at all | **2** | **3** | **4** | **5**  Very confident |
| 4 | What is your rationale behind this follow-up?  (If the follow-up has changed, please explain why) | | | | | | | | | |
