# Supplementary material for: The Impact of Expectation Management and Model Transparency on Radiologists’ Trust and Utilization of AI Recommendations for Lung Nodule Assessment on Computed Tomography: Simulated Use Study
Source: JMIR AI. 2024 Mar 13;3:e52211. doi: 10.2196/52211 (PMC11041414; doi:10.2196/52211)
Supplement: Multimedia Appendix 5 [file ai_v3i1e52211_app5.docx]

# Appendix 5 – Mental model and psychological trust

*Table 5.1: Mental model scores and psychological trust scores before and after onboarding, and at the end of the test using either informative or reflective onboarding tutorials, and either black box or explainable AI output.*

|  | | | **Mental model scores (0-11)** | | | | **Psychological trust (0-5)** | | | |
| --- | --- | --- | --- | --- | --- | --- | --- | --- | --- | --- |
|  |  |  | **Before onboarding** | **After onboarding** | | **At the end of the test** | **Before onboarding** | **After onboarding** | | **At the end of the test** |
| **Total**  (n=20) | | Mean score ± SD | 5.7 ± 2.0 | 8.6 ± 1.9 | | 8.5 ± 2.0 | 3.2 ± 0.3 | 3.3 ± 0.4 | | 3.2 ± 0.4 |
|  |  | **Differences between time points**  p-value^1^ | <0.001* | | 0.763 | | 0.619 | | 0.233 | |
| **Onboarding tutorials** | **Informative**  (n = 10) | Mean score ± SD | 5.8 ± 1.9 | 7.9 ± 1.7 | | 8.1 ± 1.4 | 3.3 ± 0.3 | 3.4 ± 0.3 | | 3.3 ± 0.4 |
|  |  | **Differences between time points**  p-value^1^ | 0.011* | | 0.317 | | 0.441 | | 0.677 | |
|  | **Reflective**  (n = 10) | Mean score ± SD | 5.6 ± 2.2 | 9.2 ± 2.0 | | 8.9 ± 2.5 | 3.2 ± 0.3 | 3.2 ± 0.4 | | 3.1 ± 0.5 |
|  |  | **Differences between time points**  p-value^1^ | 0.007* | | 0.257 | | 0.888 | | 0.089 | |
|  | **Informative versus reflective** | **Differences between changes over time**  p-value^2^ | 0.135 | | 0.156 | | 0.544 | | 0.648 | |
| **AI output** | **Black box**  (n = 10) | Mean score ± SD | 5.4 ± 1.7 | 7.9 ± 2.0 | | 7.8 ± 2.4 | 3.2 ± 0.3 | 3.3 ± 0.4 | | 3.4 ± 0.4 |
|  |  | **Differences between time points**  p-value^1^ | 0.013* | | 0.705 | | 0.236 | | 0.438 | |
|  | **Explainable**  (n = 10) | Mean score ± SD | 6.0 ± 2.3 | 9.2 ± 1.8 | | 9.2 ± 1.3 | 3.3 ± 0.2 | 3.3 ± 0.3 | | 3.0 ± 0.4 |
|  |  | **Differences between time points**  p-value^1^ | 0.007* | | 1.000 | | 0.683 | | 0.021* | |
|  | **Black box versus explainable** | **Differences between changes over time**  p-value^2^ | 0.566 | | 0.932 | | 0.288 | | 0.033* | |

*^1^ From Wilcoxon signed rank test*

*^2^ From Mann-Whitney U test*

** p < 0.05*

*Abbreviations: n, number of radiologists; SD, standard deviation.*
