# Supplementary material for: The Impact of Expectation Management and Model Transparency on Radiologists’ Trust and Utilization of AI Recommendations for Lung Nodule Assessment on Computed Tomography: Simulated Use Study
Source: JMIR AI. 2024 Mar 13;3:e52211. doi: 10.2196/52211 (PMC11041414; doi:10.2196/52211)
Supplement: Multimedia Appendix 6 [file ai_v3i1e52211_app6.docx]

# Appendix 6 – Utilization of AI recommendations

## 6.1. Changed assessments

*Table F1: Changed assessments after viewing the AI recommendations from the AI-CAD*

| **Variables** | **Changing nodule assessment** | | | **Changing malignancy probability** | | | **Changing follow-up advice** | | |
| --- | --- | --- | --- | --- | --- | --- | --- | --- | --- |
|  | Model 1 | Model 2 | Model 3 | Model 1 | Model 2 | Model 3 | Model 1 | Model 2 | Model 3 |
| Reflective onboarding | -0.043  (0.563) | -0.050  (0.497) | -0.066  (0.455) | 0.000 (1.000) | 0.003  (0.981) | 0.000  (1.000) | 0.086  (0.125) | 0.084  (0.140) | 0.085  (0.215) |
| Explainable AI | -0.071  (0.335) | -0.085  (0.246) | -0.112  (0.259) | -0.086  (0.439) | -0.080  (0.481) | -0.070  (0.635) | 0.000 (1.000) | -0.003  (0.958) | -0.047  (0.535) |
| AI found exclusively the same nodules as the radiologist | NI | -0.245*  (0.003) | -0.437*  (0.001) | NI | 0.097  (0.247) | -0.041  (0.770) | NI | -0.053  (0.376) | -0.115  (0.238) |
| AI found different nodules than the radiologist AND the radiologist consequently changed the #reported nodules | ** | ** | ** | ** | ** | ** | ** | ** | ** |
| CT scan and radiologists’ characteristics | NI | NI | Included | NI | NI | Included | NI | NI | Included |
| Intercept | 0.25  (0.000) | 0.061  (0.497) | -0.297  (0.345) | 0.271  (0.005) | 0.346  (0.003) | -0.261  (0.559) | 0.043  (0.376) | 0.002  (0.975) | -0.286  (0.234) |

*Results of the multilevel regression analysis showing the regression coefficients and p-values.*

** p < 0.05*

*** Omitted because of collinearity*

*Abbreviations: NI, not included.*

## 6.2. Changed confidence

*Table F2: Changed confidence in assessments after viewing the AI-CAD recommendations.*

| **Variables** | **Changing confidence nodule assessment** | | | **Changing confidence malignancy probability** | | | **Changing confidence follow-up advice** | | |
| --- | --- | --- | --- | --- | --- | --- | --- | --- | --- |
|  | Model 1 | Model 2 | Model 3 | Model 1 | Model 2 | Model 3 | Model 1 | Model 2 | Model 3 |
| Reflective onboarding | -0.057  (0.654) | -0.057  (0.648) | 0.012  (0.915) | 0.057  (0.575) | 0.053  (0.517) | 0.039  (0.607) | -0.114  (0.135) | -0.136  (0.080) | -0.075  (0.434) |
| Explainable AI | 0.114  (0.370) | 0.114  (0.366) | 0.032  (0.807) | 0.143  (0.161) | 0.167*  (0.044) | 0.207*  (0.016) | 0.114  (0.135) | 0.118  (0.127) | 0.042  (0.692) |
| AI found exclusively the same nodules as the radiologist | NI | -0.036  (0.727) | 0.054  (0.755) | NI | -0.130  (0.184) | 0.111  (0.455) | NI | 0.064  (0.444) | -0.056  (0.688) |
| AI found different nodules than the radiologist AND the radiologist consequently changed the #reported nodules | NI | 0.021  (0.838) | 0.084  (0.464) | NI | 0.369*  (0.000) | 0.283*  (0.001) | NI | 0.277*  (0.021) | 0.154  (0.228) |
| CT scan and radiologists’ characteristics | NI | NI | Included | NI | NI | Included | NI | NI | Included |
| Intercept | 0.557  (0.000) | 0.546  (0.001) | 0.141  (0.746) | 0.257  (0.004) | 0.425  (0.000) | -0.088  (0.777) | 0.2  (0.003) | 0.515  (0.001) | 0.000  (1.000) |

*Results of the multilevel regression analysis showing the regression coefficients and p-values.*

** p < 0.05*

*Abbreviations: NI, not included.*
